# Zebrafish Ext2 is necessary for Fgf and Wnt signaling, but not for Hh signaling

**DOI:** 10.1186/1471-213X-11-53

**Published:** 2011-09-05

**Authors:** Sabine Fischer, Beata Filipek-Gorniok, Johan Ledin

**Affiliations:** 1 European Molecular Biology Laboratory (EMBL), Meyerhofstrasse 1, 69117 Heidelberg, Germany; 2 Department of Medical Biochemistry and Microbiology, Uppsala University, Husarg. 3, 751 23 Uppsala, Sweden; 3 Department of Organismal Biology, Science for Life Laboratory, Uppsala University, Norbyv. 18A, 752 36 Uppsala, Sweden

**Keywords:** Zebrafish, Ext2, HSPG, Heparan, Fgf, Wnt, Hh

## Abstract

**Background:**

Heparan sulfate (HS) biosynthesis is tightly regulated during vertebrate embryo development. However, potential roles for HS biosynthesis in regulating the function of paracrine signaling molecules that bind to HS are incompletely understood.

**Results:**

In this report we have studied Fgf, Wnt and Hedgehog (Hh) signaling in *ext2 *mutants, where heparan sulfate content is low. We found that Fgf targeted gene expression is reduced in *ext2 *mutants and that the remaining expression is readily inhibited by SU5402, an FGF receptor inhibitor. In the *ext2 *mutants, Fgf signaling is shown to be affected during nervous system development and reduction of Fgf ligands in the mutants affects tail development. Also, Wnt signaling is affected in the *ext2 *mutants, as shown by a stronger phenotype in *ext2 *mutants injected with morpholinos that partially block translation of Wnt11 or Wnt5b, compared to injected wild type embryos. In contrast, Hh dependent signaling is apparently unaffected in the *ext2 *mutants; Hh targeted gene expression is not reduced, the Hh inhibitor cyclopamine is not more affective in the mutants and Hh dependent cell differentiation in the retina and in the myotome are normal in *ext2 *mutants. In addition, no genetic interaction between *ext2 *and *shha *during development could be detected.

**Conclusion:**

We conclude that *ext2 *is involved in Fgf and Wnt signaling but not in Hh signaling, revealing an unexpected specificity for *ext2 *in signaling pathways during embryonic development. Thus, our results support the hypothesis that regulation of heparan sulfate biosynthesis has distinct instructive functions for different signaling factors.

## Background

Heparan Sulfate Proteoglycans (HSPG) consist of proteoglycan core proteins to which long linear heparan sulfate (HS) chains carrying sulfate groups in different positions are attached [1-3]. They are ubiquitous components of cell surfaces and of the extracellular matrix. Extensive biochemical and genetic studies have shown that HSPGs influence the extracellular transport and activity of paracrine signaling molecules such as Fgf, Wnt, and Hh. Several excellent reviews on the subject of the complex role of HSPGs in developmental processes have been written [4-7].

Fgf proteins are thoroughly studied HSPG binding proteins with important roles in cell migration, proliferation and differentiation during development [8]. In *Drosophila*, the functions of Fgf receptors are abnormal in embryos with defective HSPG biosynthesis [9,10]. Mice *Ugdh *mutants (*lazy mesoderm*) have reduced synthesis of HS, chondroitin sulfate and hyaluronan, strongly suggesting that glycosaminoglycans are required for Fgf signaling [11]. Moreover, a specific inhibition of HSPG biosynthesis during brain development results in defective Fgf8 function [12]. It is suggested that the HSPG Glypcan4 enhances Fgf signaling during *Xenopus *neurulation [13] and HSPG biosynthesis is shown to influence Fgf function during limb development in zebrafish [14] and lens development and lacrimal gland induction in mice [15,16].

Wnt and Hh proteins are secreted signaling morphogens with functions in numerous developmental processes. A large number of experiments in *Drosophila *have revealed a crucial role for HSPGs in regulating these functions (reviewed in [4]). In vertebrates, several studies demonstrate an essential role for the HSPG Glypican in Wnt11 signaling during gastrulation [17-19]. Decreased HSPG sulfation reduces Shh signaling in mice [20] and decreased HS polymerization is suggested to interfere with the function of Indian hedgehog (Ihh) (discussed below).

Several different mechanisms may be used by HSPGs to effect their regulation of Fgf, Wnt and Hh function. Cell surface HSPGs can serve as co-receptors for Fgf ligands [21,22] and biochemical studies have suggested that HSPGs participate in Fgf signaling by directly interacting with Fgf ligands and their receptors to form ligand-receptor complexes [23,24]. HSPGs are probably enhancing Fgf signaling by facilitating interactions of ligands and receptors or by stabilizing the signaling complex [25]. Experiments in *Drosophila *suggest that HSPGs also act as co-receptors for the binding of Hh and Wg/Wnt to the receptors Patched and Frizzled respectively [26-29], in a similar way to the function of HSPGs in Fgf signaling. However, many observations support models where HSPGs are instead required for proper tissue distribution of Wg/Wnt and Hh proteins [30-32]. HSPGs might protect Hh and Wg/Wnt protein from degradation and increase the local concentration by reducing the dimensionality of ligand diffusion from three to two dimensions, thereby increasing the concentration of Wnt and Hh ligands close to cell surfaces (reviewed in [4]). Both Hh and Wnt proteins are lipid modified and they are unlikely to freely diffuse between cells. Instead, their movement probably involves additional molecules such as lipoprotein particles [33]. Interestingly, a recent study suggests that HSPGs can influence lipid-linked morphogen signaling by a direct interaction with lipoprotein particles [34].

The exostosin gene family contains glycosyltransferases required for HS biosynthesis, and include, in mouse, *Ext1, Ext2, Extl1, Extl2 *and *Extl3*. During HS biosynthesis in the Golgi compartment, a serine residue in the proteoglycan core protein is modified by stepwise addition of monosaccharides to form a linkage tetrasaccharide which constitutes the substrate for Ext enzymes to initiate and polymerize the HS polysaccharide chain (reviewed in [3]). Ext1 and Ext2 together form a copolymerase which is responsible for the polymerization process where repeating units of *N*-acetylglucosamine and glucuronic acid are incorporated in the growing linear polysaccharide chain. During polymerization, the HS chains are modified by the addition of sulfate groups in tissue-specific patterns [35,36]. The relative expression of HSPG biosynthesis genes may sometimes determine the outcome of the process, but the regulation of HSPG biosynthesis is far from understood [37-39]. *Drosophila *exostosins genes include *ttv*, *sotv *and *botv *(encoding homologues of mouse *Ext1*, *Ext2 *and *Extl3*, respectively), which have been demonstrated to be important for the signaling activity of Hh and Wg/Wnt and for shaping extracellular morphogen gradients (reviewed by [4]). Ext1-null and Ext2-null mice both fail to gastrulate, pointing to the early essential roles for *Ext *genes in developing embryos [40,41]. Reduced expression of Ext1 results in delayed hypertrophic differentiation and endochondral ossification of the chondrocytes of limb growth plates, probably caused by increased Ihh diffusion [42,43]. Mutations in *Ext *genes is the cause of the human disease Multiple Hereditary Exostoses (MHE) [44,45].

The zebrafish *ext2 *and *extl3 *genes are ubiquitously expressed during zebrafish development and they are disrupted in *dackel (dak) *and *boxer *(*box*) mutants, respectively [46]. In *ext2 *and *extl3 *mutants, maternally deposited mRNA provides sufficient levels of HSPGs to allow normal gastrulation of the embryo while HS polymerization is subsequently reduced in all tissues [46]. *ext2 *and *extl3 *mutants were originally isolated based on their defective limb development [47] and we have previously shown that Fgf10 signaling during limb development requires *ext2 *and *extl3 *[14]. *ext2 *and *extl3 *mutants also show defects in cartilage and pharyngeal arch morphogenesis [48,49], development of the ear [50], and axon sorting in the optic tract [46,51].

The role of *ext2 *in regulating paracrine signaling is of specific interest for understanding the molecular mechanisms of the human disease MHE, but is also of general interest for elucidating the role of the HS portion of the HSPGs, as opposed to the functions of the proteoglycan core protein. In this study we systematically investigate the general role of *ext2 *in FGF, Wnt and Hh signaling during zebrafish tissue patterning and organogenesis. We find that Fgf signaling is generally reduced in *ext2 *mutants, based on the reduced expression of target genes and the observation that remaining expression of Fgf target genes is sensitized for treatment with a pharmacological Fgf inhibitor, suggesting that all Fgf signaling is dependent on *ext2 *function. We also found that a smaller decrease of Wnt11 and Wnt5b translation is required in *ext2 *mutants, compared with siblings, to interfere with Wnt dependent processes, indicating that *ext2 *is also involved in Wnt signaling. In contrast, we find that Hh signaling does not act on the same pathways as *ext2*. We base this conclusion on the observation that Hh signaling in *ext2 *mutants (1) induces normal expression of target genes, (2) functions in Hh signaling dependent cell differentiation and (3) is not sensitized by partial inhibition of Hh signaling compared to control embryos. We propose that the *ext2 *gene, as well as any gene influencing levels of HS in zebrafish tissues, is a candidate for regulation of aspects of Fgf and Wnt function, while Hh signaling is likely to be largely independent of *ext2 *function.

## Results

### Expression of the Fgf signaling target gene *etv5b *is reduced in *ext2 *mutants

**Figure 1:**
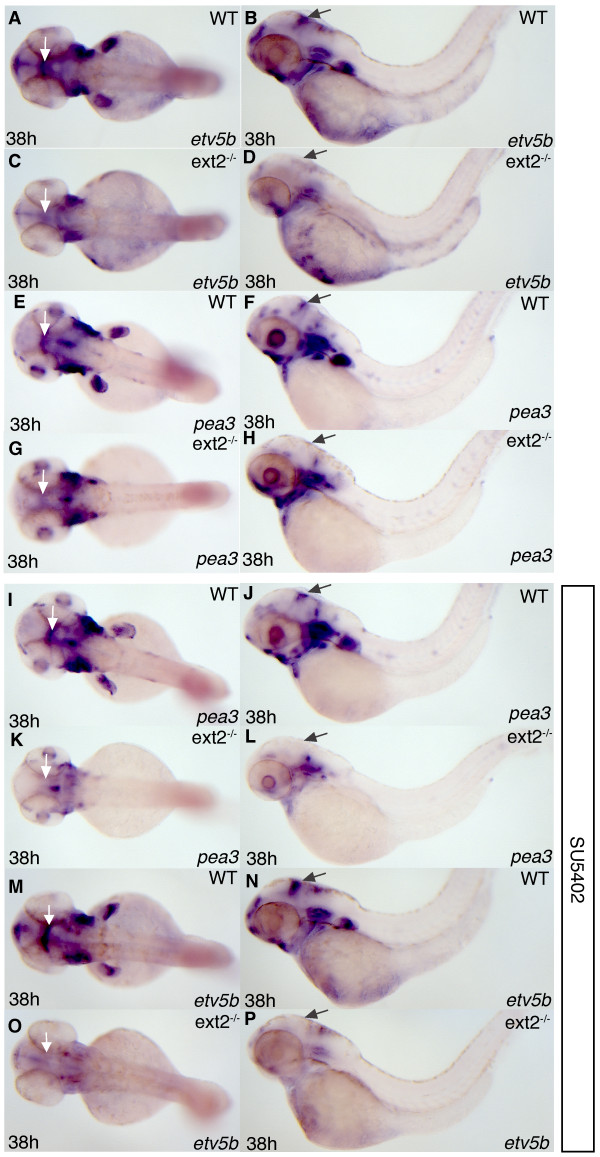
***etv5b *and *pea3 *expression is reduced in ext2 mutants**. Lateral view (left panels) and dorsal view (right panels) of *etv5b *(A-D, M-P) and *pea3 *(E-L) expression in 38 hpf *ext2 *mutants (C, D, G, H, K, L, O, P) and WT embryos (A, B, E, F, I, J, M, N). (**I-P**) Embryos treated with 8 μM SU5402 3 h prior to fixation. Note that *pea3 *expression in WT embryos is only marginally affected by the inhibitor (compare E-F with I-J) while *pea3 *expression is almost completely inhibited by the same treatment in *ext2 *mutants (compare G-H with K-L). The arrows label the MHB.

**Figure 2:**
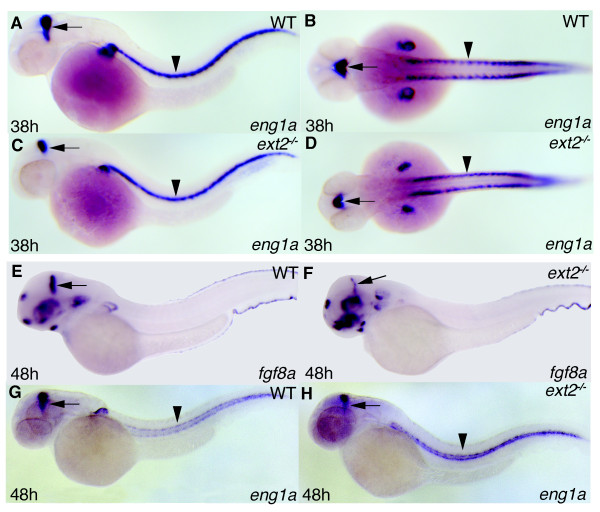
**Reduced expression of *eng1a *and *fgf8a *in MHB but normal *eng1a *expression in muscle pioneers**. Lateral view (A, C, E-H) and dorsal view (B, D) of 38 hpf (A-D) and 48 hpf (E-H) embryos. Expression of *eng1a *and *fgf8a *in *ext2 *mutants (C, D, F, H) and WT embryos (A, B, E, G). The arrows label the MHB and arrowheads label muscle pioneer cells.

The zebrafish *ext2 *mutant only contains a fraction of the normal HS levels in its tissues at 24 hpf [46] but even though Fgf signaling is believed to depend on HSPGs, Fgf10 function in the developing limb is so far the only defective Fgf signaling activity observed in *ext2 *mutants [14]. Is any Fgf signaling, other than that of Fgf10, independent of *ext2 *function during embryonic development? We decided to test the role of *ext2 *in Fgf signaling by investigating the transcription of Fgf target genes. In zebrafish, expression of the transcription factor *etv5b (erm) *is a direct readout of Fgf signaling [52,53]. We therefore crossed heterozygous *ext2 *individuals and examined activation of *etv5b *in the offspring at different times of embryonic development. At 15 hpf and 24 hpf, all embryos displayed normal expression of *etv5b *(data not shown). However, at 38 hpf (Figure 1A-D), and at 48 hpf and 60 hpf (data not shown), *etv5b *expression in *ext2 *mutants is clearly reduced at all sites of expression compared to control embryos. In particular, the expression in the midbrain-hindbrain boundary is almost eliminated while some *etv5b *expression remains in the branchal arches, in the otic vesicle and in diencephalon. The reduction of Fgf signaling does not appear to be caused by reduced expression of Fgf protein, since *fgf4*, *fgf10 *and *fgf24 *expression remains as high in *ext2 *mutants as in control embryos at 38 hpf in all tissues except the developing limb (additional file 1A-F). Here the defects in Fg10 signaling have stopped the outgrowth [14]. Also, at 38 hpf *fgf8a *expression is comparable in *ext2 *mutants and control embryos except in the limbs (additional file 1G-H), although *ext2 *mutants display an increased prevalence of reduced fgf8a expression in the midbrain-hindbrain boundary (MHB) from 38 hpf and onwards (discussed below; Figure 2E-F). We conclude that expression of the Fgf signaling target gene *etv5b*, but not expression of Fgf proteins, is generally decreased in *ext2 *mutants.

### *ext2 *is a general enhancer of Fgf target gene expression

The transcription factor *pea3 *is, like *etv5b*, dependent on Fgf signaling for its expression [52,53]. Surprisingly, with the exception of a reduction in midbrain-hindbrain boundary expression, *pea3 *expression is, in contrast to *etv5b *expression, largely similar in control embryos and *ext2 *mutants (compare panels E-F and G-H in Figure 1). Does this mean that some Fgf signaling, which predominantly induces *pea3 *expression rather than *etv5b *expression, is in fact independent of *ext2 *function? This was tested by exposing the embryos to the Fgf signaling inhibitor SU5402 which blocks both *etv5b *and *pea3 *expression [52,53]. We reasoned that if *pea3 *expression in *ext2 *mutants was the result of HS independent fgf signaling, it should be equally sensitive to treatment with SU5402 as control embryos. However, while exposure of 8 mM SU5402 3 h prior to fixation only marginally reduces *pea3 *expression in 38 hpf control embryos (Figure 1I-J), *pea3 *expression in *ext2 *mutants is nearly eliminated by the same treatment. (Figure 1K-L). A similar difference in sensitivity was observed between 48 hpf and 60 hpf *ext2 *mutants and control embryos (data not shown). Moreover, the weak *etv5b *expression in *ext2 *mutants (Figure 1C-D) was blocked by 8 mM SU5402 exposure (Figure 1O-P), while *etv5b *expression was only mildly affected in SU5402 treated control embryos (Figure 1M-N) compared to non-treated control embryos (Figure 1A-B). We conclude that no expression of the Fgf signaling target genes *etv5b *and *pea3 *is independent of *ext2 *in the developing zebrafish embryo.

### Impaired brain patterning in *ext2 *embryos

Based on the reduced expression of Fgf target genes in *ext2 *mutants (Figure 1A-H), we hypothesized that not only is Fgf10 signaling defective in the developing limb [14] but that *ext2 *is enhancing Fgf signaling in general. To test this hypothesis we investigated the function of Fgf signaling in tissues other than the developing limb.

The midbrain-hindbrain boundary (MHB) is important for the patterning of the vertebrate brain and Fgf protein expressed in the MHB, in particular Fgf8a but not Fgf10, fulfill crucial organizing functions [54]. Lee and co-workers have reported normal *fgf8a *expression in MHB of *ext2 *mutants at 24 hpf [46] and since *fgf8a *is known to regulate its own expression in MHB [55], they concluded that Fgf signaling during early brain patterning was normal. However, the reduced expression of *etv5b *and *pea3 *in MHB at 38 hpf in *ext2 *mutants compared to control embryos (Figure 1A-H) indicates that Fgf signaling in MHB at later stages of development is reduced. We then compared the expression of two marker genes for MHB signaling, *eng1a *and *fgf8a*, in *ext2 *mutants and control embryos. At 38 hpf a majority of *ext2 *mutants (60%, n = 14) display reduced expression of *eng1a *in MHB (Figure 2A-D) while in contrast a majority (76%, n = 17) of 38 hpf *ext2 *mutants express normal levels of *fgf8a *in MHB (additional files 1G-H). However, at 48 hpf all *ext2 *mutants express reduced levels of *eng1a *(2G-H) and *fgf8a *(Figure 2E-F). Thus, a portion of *ext2 *mutants express reduced levels of *eng1a *and *fgf8a *at 38 hpf and the reduction is fully penetrant at 48 hpf. Notably, the discovery that the reduction in *etv5b *and *pea3 *expression in the MHB of *ext2 *mutants (Figure 1A-H) precedes the fully penetrant reduction of *eng1a *and *fgf8a *expression (Figure 2E-H) suggests that a reduction of Fgf signaling is a possible cause of the reduction in MHB signaling. We conclude that although early brain patterning is normal, *ext2 *mutants show a gradually increased prevalence of abnormal brain patterning at later stages, consistent with a decline in Fgf signaling in the MHB after 24 hpf.

### *ext2 *interacts genetically with Fgf signaling during tail development

**Figure 3:**
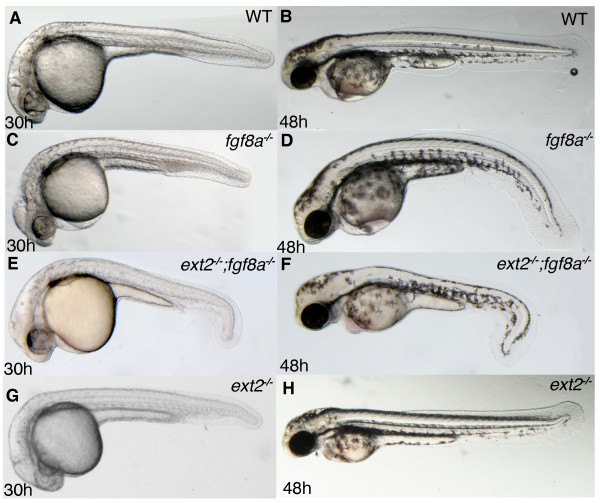
**Tail development is disturbed in *ext2;fgf8a *double mutants**. Lateral views of 30 hpf (left panels) and 48 hpf (right panels) embryos. (A, B) WT embryos, (C, D) *fgf8a *mutants and (E, F) *fgf8a;ext2 *double mutants. Tail morphology of *ext2 *mutants are indistinguishable from that of WT embryos at 30 hpf and 48 hpf (not shown).

Fgf signaling is crucial for the development of the trunk and tail [56] and *fgf8a *and *fgf24 *act together to promote zebrafish tail development [57]. We crossed the *ext2^tw25e ^*allele into the *fgf8a^ace ^*mutant background. In the offspring (n = 230) of *ext2^+/-^;fgf8a^+/- ^*parents, 23% of the progeny was identified as *fgf8a *mutants at 24 hpf based on absence of the midbrain-hindbrain boundary [58]. Interestingly, 27% of this selection displayed a distinct "hooked tail" morphology at 30 hpf (Figure 3C) and at 3 dpf we identified them as *ext2;fgf8a *double mutants as they never developed pectoral fins. The hooked tail morphology of *ext2;fgf8a *double mutants is fully penetrant from 30 hpf until the mutants die at 4-6 dpf (Figure 3E-F and data not shown) and is never observed in *fgf8a *mutants, which develop a slightly curved body axis at 30 hpf (Figure 3C). The morphology of the caudal fin in *ext2 *mutants remain indistinguishable from control embryos suggesting that the hooked tail phenotype seen in *ext2;fgf8a *double mutants is not an additive effect of two independent tail phenotypes, but instead the result of a genetic interaction between *fgf8a *and *ext2*. In addition, blocking Fgf24 translation in *ext2 *mutants by injecting 5 ng MO2-fgf24 [59] results in a similar hooked tail phenotype as *ext2;fgf8a *double mutants (data not shown). These observations suggest that *ext2 *is involved in Fgf signaling during tail development and support - together with the observed reduction of Fgf signaling in the MHB and the reduced expression of Fgf signaling target genes in *ext2 *mutants (described above) - the hypothesis that *ext2 *has a general function in Fgf signaling in zebrafish after 24 hpf.

### *ext2 *has a role in Wnt signaling during zebrafish development

**Figure 4:**
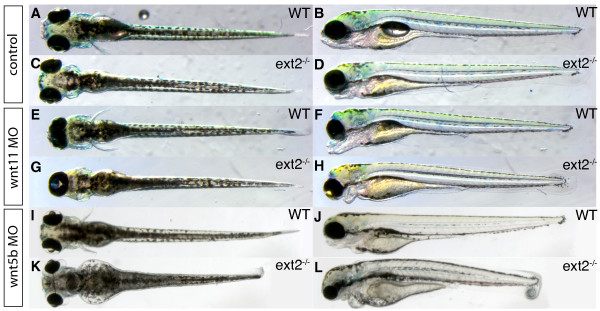
***ext2 *mutants are sensitive to a partial reduction in Wnt11 and Wnt5b translation**. Dorsal view (left panels) and lateral view (right panels) of 3 dpf WT embryos (A-B, E-F, I-J) and *ext2 *mutants (C-D, G-H, K-L). Embryos injected with 10 ng MO1-wnt11 (E-H) and embryos injected with 7 ng MO1-wnt5b (I-L). Note that injection of the morpholinos results in a stronger phenotype in the *ext2 *mutants compared to WT embryos.

Has *ext2 *also a role in Wnt signaling during zebrafish embryonic development? We reasoned that if *ext2 *acts as an enhancer of Wnt signaling, *ext2 *mutants with their general reduction in HS content should be more sensitive to a partial reduction in the translation of Wnt ligands compared to control embryos. The *wnt11 (silberblick) *mutant has a characteristic partial fusion of the eyes due to reduced convergent-extension cell movements which impairs anterior movements of the prechordal plate and the ventral forebrain [60]. Injections of 10 ng MO1-wnt11 into control embryos have been shown to phenocopy the mutation [61]. We tested whether *ext2 *was involved in *wnt11 *function by injecting 7 ng MO1-wnt11 in the offspring (n = 210) of adult *ext2^+/- ^*individuals. In control embryos the injection typically resulted in a characteristic *wnt11 *mutant phenotype with a slight fusion of the eyes (Figure 4E-F). In contrast, injected *ext2 *mutants, identified by their lack of pectoral fins, typically developed a stronger phenotype and 51% of *ext2 *mutants were in fact cyclopic (Figure 4G-H), which was only observed in one control embryo. In an analogous experiment we tested the effect of reducing Wnt5b translation in *ext2 *mutants and siblings. Injection of 14 ng of wnt5-MO1 in WT embryos phenocopied the reduction in body length and the hammerhead phenotype characteristic for the *wnt5b *(*pipe tail) *mutant [61-63]. When the offspring (n = 128) of adult *ext2^+/- ^*individuals were injected with 7 ng MO1-Wnt5b, the *ext2 *mutants typically phenocopied the *wnt5b *mutant head and body length phenotype (Figure 4K-L), while the morphology of the injected siblings were almost WT like (Figure 4I-J). Thus, in *ext2 *mutants a smaller reduction of Wnt11 and Wnt5b translation is sufficient to specifically interfere with Wnt function compared to siblings. This indicates that *ext2 *acts in Wnt signaling pathways in zebrafish.

### *ptc1 *expression and Hh dependent cell differentiation in the zebrafish retina and myotome are normal in *ext2 *mutants

**Figure 5:**
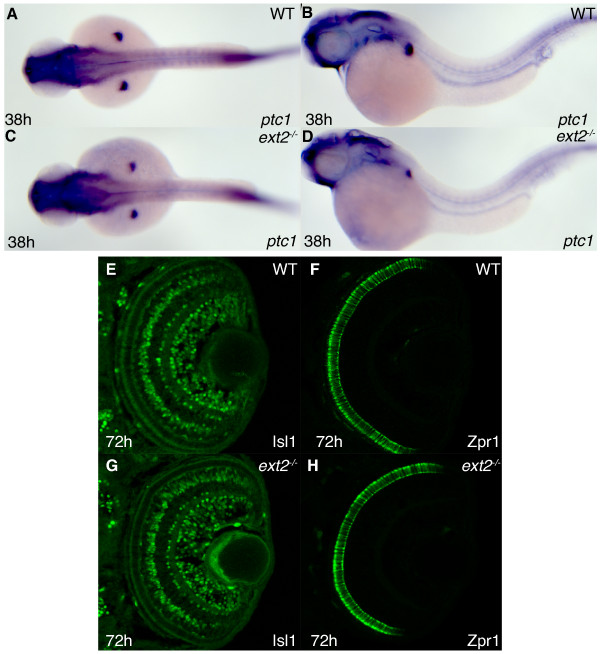
**Hh signaling in *ext2 *mutants elicits normal *ptc1 *expression and functions normally in differentiation of cells in the retina**. Lateral view (A, C) and dorsal view (B, D) of *ptc1 *expression in 38 hpf WT embryos (A, B) and *ext2 *mutants (C-D). Asterisks label the developing limbs. The difference in somite staining between A-B and C-D is within the range of individual variation (also see additional figure 3A-B) (E-H) Confocal sections of the retina at 72 hpf, with anterior to the top. Detection of the Isl1 protein (E, G) and Zpr1 protein (F, H) in WT retinas (E-F) and *ext2 *mutant retinas (G-H) reveal normal Hh signaling in *ext2 *mutants during patterning of the zebrafish retina.

Lee and co-workers have reported that expression of the Hh receptor *ptc1*, which is induced in response to Hh signaling, is normal during early brain development in *ext2 *mutants [46]. We investigated the function of Hh signaling after 24 hpf, when immunohisochemical staining of HS shows that nothing but traces of HS polysaccharides remain in *ext2 *mutant tissues [46] and data not shown). In contrast to expression of the Fgf signaling target gene *etv5b *(Figure 1A-D), *ptc1 *is normally expressed in *ext2 *mutants at 38 hpf (Figure 5A-D) indicating normal Hh function. The reduced *ptc1 *expression in the pectoral fin (Figure 5. C-D) is caused by a reduction in Fgf10 signaling capacity [14] and not by defective Hh signaling [64]. We next asked whether Hh signaling correctly induces cell differentiation in *ext2 *mutants during later stages of development. *shha (syo) *expression between 32-37 hpf drives a wave of neurogenesis across the retina and in *shha *mutants the retinal tissue is poorly differentiated and highly disorganized [65]. Expression of the proteins Isl1 and Zpr1 mark distinct cell populations which are all severely reduced in the retina of *shha *mutants at 72 hpf [66]. When *ext2 *mutants were studied, we found that retinal cell differentiation in these embryos is indistinguishable from that in control embryos where the retina develops normal cellular layers (Figure 5 E-H). Another well studied function of Hh signaling is in cell differentiation in the zebrafish myotome where muscle cell differentiation is regulated by Hh signaling [67]. *eng1a *expression in muscle pioneers in the myotome can be regarded as a readout for Hh signaling [68] and *eng1a *expression in the myotome is similar in *ext2 *mutants and siblings from segmentation to hatching (Figure 2A-D, G, H and data not shown). We therefore conclude that Hh signaling induces normal expression of *ptc1 *in *ext2 *mutants and that Hh dependent cell differentiation in the retina and the myotome is normal.

### In contrast to Fgf and Wnt signaling, Hh signaling is not sensitized in *ext2 *mutants

We next investigated whether Hh signaling in *ext2 *mutants is more susceptible to a reduction in Hh expression than in control embryos, in a similar way to the results presented above for Fgf and Wnt signaling. Nasevisius and co-workers have reported that inhibiting Hh function in zebrafish embryos by injecting a *shha (sonic you) *translation blocking morpholino (MO1-shha) results in embryos with U-shaped somites [69] which phenocopies the somite phenotype of the *shha *mutants [70]. We injected 14 ng MO1-shha morpholino in offspring (n = 153) derived from crossings of heterozygous *ext2^+/- ^*individuals, and we scored the presence of U-shaped somites in *ext2 *mutants at 3 dpf (additional file 2A-D). However, MO1-shha injected *ext2 *mutants displayed a similar proportion of individuals with U-shaped somites as siblings (47% and 43%, respectively). Repeating the experiment with 7 ng of MO1-shha morpholino (n = 201) resulted in fewer individuals with U-shaped somites but the proportion of *ext2 *mutants and siblings with U-shaped somites was still similar (14% and 11%, respectively). Even a lower concentration of MO1-shha morpholino (3, 5 ng) did not result in any individuals with U-shaped somites. Taken together, these data indicate that the MO1-shha is equally effective in inducing U-shaped somites in *ext2 *mutants as in siblings.

In another experiment we tested Hh signaling in *ext2 *mutants for its sensitivity to cyclopamine, a drug which blocks Hh signaling by direct binding to the Hh signal transduction component Smoothened [71]. If Hh signaling was less robust as a result of lowered HS content in *ext2 *mutants, it should be possible to block the expression of the Hh signaling downstream target *ptc1 *with a lower concentration of cyclopamine in *ext2 *mutants than in the siblings. However, when we treated the offspring (n = 100) from crossings between adult heterozygous *ext2 *mutant carriers with 50 μM cyclopamine 32-38 hpf, *ptc1 *expression was similarly reduced at 38 hpf in both *ext2 *mutants (identified by their smaller limb buds) and siblings (additional file 2E-H). Repeating this experiment with 25 μM cyclopamine did not result in an apparent reduction in *ptc1 *expression in either *ext2 *mutants or siblings and taken together these data indicate that cyclopamine is equally effective in reducing *ptc1 *expression in *ext2 *mutants and siblings. We finally tested whether *ext2 *and *shha *interact genetically by crossing the *ext2^tw25e ^*allele into the *shha^t4 ^*background. To reduce the *shha *gene dose in *ext2 *mutants we crossed heterozygous *ext2^+/-^;shha^+/- ^*double carriers with *ext2^+/- ^*carriers resulting in 50% of *ext2 *mutants lacking one functional *shha *gene. We reasoned that if the reduced gene dose of *ssha *in *ext2 *mutant did affect hedgehog signaling, then 50% of the isolated *ext2 *mutants in this cross would display changes in *ptc1 *expression. However, *ptc1 *expression was indistinguishable in all *ext2 *mutants at 38 hpf (n = 34, additional file 3A) and 48 hpf (n = 23, additional file 3B) indicating that one or two copies of a functional *shha *gene did not affect hedgehog signaling in *ext2 *mutants.

We conclude that reducing Hh signaling in *ext2 *mutants by several different methods does not affect expression of Hh signaling target genes or the severity of morphological phenotypes associated with defective Hh signaling. This is in strong contrast to Fgf and Wnt signaling. Taken together these data suggest that *ext2 *differently modulates the function of HS binding paracrine signaling factors.

## Discussion

In this study we present evidence for differences in the role of *ext2 *in Fgf, Wnt and Hh signaling during zebrafish embryo development. While *ext2 *functions as an enhancer of Fgf and Wnt signaling, Hh signaling is apparently not affected by the decreased HS levels caused by the *ext2 *deficiency.

### Fgf signaling in *ext2 *mutants

We previously reported that *ext2 *is required for Fgf10 signaling but not for Fgf24 or Fgf4 signaling during limb bud development, which suggested that different Fgfs have specific requirements for HSPGs *in vivo *[14]. In this study we addressed the follow up question of whether *ext2 *has a general role in Fgf signaling. We show that the two Fgf signaling target genes *pea3 *and *etv5b *are reduced in tissues other than the developing limb and that all remaining expression in the mutants is sensitised for inhibition of Fgf signaling. For example, in the MHB where Fgf10 is not expressed, *pea3 *and *etv5b *expression are reduced in *ext2 *mutants at 38 hpf (Figure 1C, D, G, H). Fgf8 is a key mediator of MHB signaling and mice with the conditionally disrupted *Ext1 *gene, encoding one component of the HS copolymerase complex EXT1/EXT2 [3], exhibit reduced HS polymerization in the brain and display similar abnormal expression of Fgf and Engrailed proteins [12] as *ext2 *mutants (Figure 2F, H). MHB expression of *etv5b *and *pea3 *is reduced in *ext2 *mutants at 38 hpf (Figure 1D, H) but marker genes of MHB signaling such as *fgf8a *and *eng1a *are not generally reduced until 48 hpf (Figure 2E-H), suggesting that a reduction in Fgf signaling precedes and possibly causes the reduction in MHB signaling at 48 hpf. Notably, the patterning of the MHB contains several feedback loops (reviewed in [54]) and the reduced Fgf signaling in MHB of *ext2 *mutants (Figure 1A-H) might well cause a complex mispatterning of this tissue. Notably, although *eng1a *expression is subsequently reduced in the MHB, it is never absent (Figure 5 and data not shown) indicating that the MHB retains some signaling activity during embryo development. Moreover, in zebrafish, both Fgf8 and Fgf24 are required to promote posterior mesodermal development. Removal of both gene functions significantly impairs development of posterior tissues [57]. The tail development in *ext2 *mutants is disturbed when either Fgf24 or Fgf8 is removed (Figure 3E, F) which suggests a role for *ext2 *in maintaining Fgf signaling pathways. Taken together, our data suggest that *ext2 *acts as a general enhancer of Fgf signaling after 24 hpf.

### Mechanism of reduction of Fgf signaling in *ext2 *mutant

So, by which mechanism is Fgf signaling reduced in *ext2 *mutants? HS binds to both Fgf ligands and receptors and facilitates receptor dimerization [72]. In the most straightforward model, the reduced level of HS in the *ext2 *mutants would result in the formation of fewer signaling Fgf receptor dimers. However, the observation that *etv5b *expression is more greatly reduced than *pea3 *expression (Figure 1A-H), suggests more complex mechanisms. During zebrafish development, *etv5b *is expressed further away from cells expressing Fgf ligands than *pea3*, suggesting that cells typically require less Fgf ligands to elicit *etv5b *than *pea3 *expression [53]. Therefore, if the only mechanism for reduction of Fgf signaling in *ext2 *mutants was a decreased participation of HS in ligand/receptor complexes, then *pea3 *expression would be expected to be more reduced than *etv5b *expression. In fact, the opposite was observed (Figure 1A-H). A role for HSPGs in Fgf transport, as has previously been described for Wnt and Hh transport [4], could possibly explain this result. If HS participates in Fgf transport, then cells positioned at greater distances from Fgf expressing cells, such as many *etv5b *expressing cells, would be exposed to a lower concentration of Fgf ligands, resulting in the observed effect in *etv5b *expression (Figure 1A-D). In contrast, *pea3 *is normally only expressed in cells close to Fgf expressing cells and would be less affected by reduced Fgf transport (Figure 1E-H). It should also be remembered that HSPGs, as well as Fgf receptors and ligands, interact with a large number of extracellular molecules [73] and the mechanisms by which *ext2 *enhances Fgf signaling might turn out to be complex.

### Wnt signaling in *ext2 *mutants

In *Drosophila*, *sotv (ext2) *and other *ext *genes are necessary for normal Wt function during wing development [4], but no study has yet reported a role for vertebrate *ext *genes in Wnt signaling. However, indirect evidence suggests that HS biosynthesis is necessary for vertebrate Wnt function since the HSPG glypican is required for vertebrate Wnt signaling [17-19]. Topoczewski and co-workers have shown that the zebrafish glypican *gpc4 (knypek) *potentiates non-canonical Wnt signaling and removal of *gpc4 *in the *wnt11 *mutant *silberblick *(*slb*) is correlated with defective anterior extension of midline cells, leading to failure of eye field separation and cyclopia. However, the glypican core protein can promote paracrine signaling independent of HS modification [74]. Our results demonstrate that inhibition of Wnt11 translation in *ext2 *mutants phenocopies (Figure 4E-H) the *gpc4^-/-^*;*wnt11^-/- ^*double mutant eye phenotype [17] suggesting that it is indeed the HS modification of *gpc4 *that exhibit the Wnt signaling promoting function. We also studied the function of *wnt5b *in *ext2 *mutants. Wnt5b is known to physically interact with the the zebrafish glypican *gpc3 *[19] and the *wnt5b *mutant *pipetail *(*ppt*) displays defects in axis elongation and cartilage differentiation which can be phenocopied by injection of a morpholino that blocks the Wnt5b translation [61]. We found that a partial reduction of Wnt5b translation, insufficient to interfere with Wnt5b function in control embryos (Figure 4 I-J), still elicits the shorter body axis and hammerhead-like phenotype of *ppt *mutants [61] when injected in *ext2 *mutants (Figure 4K-L). Our data thus shows that *ext2 *potentiates vertebrate Wnt signaling and support the hypothesis that defects in Wnt5b function could be the explanation for the similar chondrocyte stacking phenotype in *wnt5b *and *ext2 *mutants [49].

### Hh signaling in *ext2 *mutants

Several studies have shown the requirement for *ext2 (sotv) *and other *ext *genes for proper distribution and signaling activity of Hh signaling molecules in *Drosophila *[4]. However, in vertebrates the picture is less clear. In mice, the diffusion of Ihh during bone development is affected by a reduction of EXT1 expression [42,43] but not of EXT2 [41]. Previous studies of zebrafish *ext2 *mutants have not found evidence for defective Hh signaling during early brain patterning [46], limb development [64] or chondrocyte stacking [49]. In this study we find further evidence for the lack of effects on Hh signaling in *ext2 *mutants; Hh signaling induces normal expression of the target gene *ptc1 *in *ext2 *mutants and Hh dependent cell differentiation in the retina and myotome is identical in control and *ext2 *mutants. In addition, control and *ext2 *mutant embryos respond to the same extent to reduction of Hh signaling, when Hh target gene expression and phenotypes associated with defective Hh signaling are studied. Since *ext2 *mutants have a fraction of the normal HS content in their tissues [46], we cannot exclude the possiblility that an even stronger reduction of HSPG biosynthesis would affect Hh signaling in zebrafish or even that more subtle aspects of Hh signaling are in fact altered in *ext2 *mutants. However, we conclude that, in comparison to the significant role for *ext2 *in Fgf and Wnt signaling, Hh signaling is virtually independent of *ext2 *function.

### ext2 modulates the function of specific HS binding signaling factors differently

HSPG biosynthesis is spatially and temporally varied during embryonic development [35,75,76] and numerous studies have suggested the possibility of instructive functions for HS biosynthesis in paracrine signaling (reviewed by [5]). Our results suggest that genes which, like *ext2*, affect the HS content in tissues, are far more likely to regulate Fgf and Wnt signaling than Hh signaling. The explanation for the abnormal Hh function in mice with reduced Ext1 expression [42,43] - but not in mice or zebrafish with reduced Ext2 expression ([41,46] and this study) - might be that HS polymerization is more effectively reduced by a reduction in Ext1 than Ext2. Alternatively, since relative expression levels of mouse EXT1 and EXT2 have distinct effects on subsequent HSPG sulfation [38] and Hh signaling is disturbed in mice with abnormal HS structures [20], it is possible that Hh signaling is sensitive to putative abnormal HS structure caused by EXT1 but not EXT2 deficiency. Expression of *ext2 *is ubiquitous during zebrafish development [46] but it is interesting to note that *ext1 *genes exhibits specific spatial and temporal expression [77], indicating that HS levels and structure might vary according to specific effects on Fgf, Wnt and Hh signaling.

## Conclusions

Most paracrine signaling factors bind to HSPGs and genes affecting polymerization of HS are generally assumed to have important but permissive roles for paracrine signaling. In this study we have found an unexpected specificity in how zebrafish *ext2 *affects the function of paracrine signaling factors, suggesting possible instructive functions for genes regulating HS polymerization during animal development.

## Methods

### Zebrafish lines

WIK and Tübingen were used as wild-type strains. Mutant strains used were: the *fgf8a *mutant *acerebellar *(*ace*), the *shha *mutant *sonic you (syo^t4^) *and the *ext2 *mutant *dackel *(*dak^tw25e^*). Embryos were cultured in E3 medium, with or without the addition of 0.003% 1-Phenyl-2-thiourea (PTU, Sigma) to inhibit pigmentation. Embryos were staged according to hours post fertilisation (hpf) [78]. In experiments including offspring from heterozygous adults, mutants and siblings were distinguished by previously described phenotypic characterisation: *ext2 *mutants were identified by the reduced size and signaling activity of the pectoral fin bud after 32 hpf [14,64] or by the abnormal jaw morphology after 3 dpf [48]. The *fgf8a *mutants were identified based on the absence of MHB 24 hpf [58].

### Microinjection of morpholino oligonucleotides

The following morpholino oligonucleotides (MO) were purchased from GeneTools: MO2-fgf24 5'-AGGAGACTCCCGTACCGTACTTGCC-3' [59], MO1-wnt11 5'-GAAAGTTCCTGTATTCTGTCATGTC-3' [61], MO1-wnt5b 5'-GTCCTTGGTTCATTCTCACATCCAT-3' [61], MO1-shha 5'-CAGCACTCTCGTCAAAAGCCGCATT-3' [69]. All oligonucleotides were solubilized in sterile water and injected into one-cell stage zebrafish embryos at concentrations ranging from 3,5-14 ng/embryo.

### Histochemical methods

In situ hybridisation was performed as previously described [79]. The following mRNA in situ probes were used: *eng1a *[80], *etv5b *[53], *fgf4 *[64], *fgf8a *(Reifers et al., 1998), *fgf10a *[81], *fgf24 *([59], *pea3 *[53], *ptc1 *[82]. Antibody labeling was carried out on 12 μm thick cryosections and analyzed with a Leica confocal microscope. The following antibodies were used: mouse anti-Isl1 (Developmental Studies Hybridoma Bank; 1:50) and mouse anti-Zpr1 (University of Oregon; 1:200).

### Cyclopamine and SU5402 treatement

Cyclopamine (Toronto Research Chemicals, cat# C988400) was dissolved in ethanol at 20 mM. Treatment was performed with 50 μM or 25 μM solution of cyclopamine or a corresponding control ethanol solution in E3 embryo medium. FGF signaling inhibitor SU5402 (Calbiochem, cat # 572630) was dissolved in DMSO at 8 mM. Treatment was performed with a 10 μM solution of SU5402 or a corresponding control DMSO solution in E3 embryo medium.

## Authors' contributions

SF established experimental methods and assisted during experiments. BF assisted during experiments. JL designed and performed all the experiments and drafted the manuscript. All authors read and approved the final manuscript.

## Supplementary Material

Additional file 1 **Expression of *fgf *genes in *ext2 *mutants**. Dorsal view of *fgf4 *(A-B), *fg24 *(C-D), *fgf10 *(E-F), *fgf8 *(G-H) expression in control embryos (left panels) and ext2 mutants (right panels) at 38 hpf. Note that comparable levels of all examined *fgf *genes are expressed at 38 hpf, with the exception of the developing pectoral fin. Black arrows label the presence (left panels) or absence (right panels) of developing pectoral fins. Click here for file

Additional file 2 **Hh signaling is not sensitizised in *ext2 *mutants**. Morpholino injection experiment (A-D). Lateral view of 3 dpf control (A, C) and *ext2 *mutants (B, D). In (C, D) 14 ng MO1-shha have been injected in the one-cell stage which has resulted in U-shaped somites in a portion of injected embryos (see result section). The shape of the somites is emphasized in the right panel (A-D). Cyclopamine treatment experiment (E-H). *ptc1 *expression in 38 hpf embryos subjected to 50 μM cyclopamine 32-38 hpf (E-H). Dorsal view (E, F) and dorsolateral view (G-H). WT embryos (E, G) and *ext2 *mutants (F-H). Click here for file

Additional file 3 **Hh signaling is not reduced in *ext2^-/-^;shha^+/- ^*mutants**. Genetic interaction experiment (A-B). Lateral view of *ptc1 *expression in 38 hpf (A) and 48 hpf (B) embryos from crossings of heterozygous *ext2^+/-^;shha^+/- ^*double carriers with *ext2^+/- ^*single carriers. Siblings (left row of embryos) and *ext2^-/-^*or *ext2^-/-^;shha^+/- ^*mutants (right row of embryos). Arrows label the position of the pectoral fin. Click here for file
