# Supplementary figures and images for: Zebrafish Ext2 is necessary for Fgf and Wnt signaling, but not for Hh signaling
Source: BMC Dev Biol. 2011 Sep 5;11:53. doi: 10.1186/1471-213X-11-53 (PMC3183004; doi:10.1186/1471-213X-11-53)

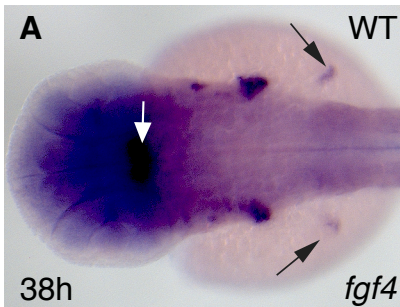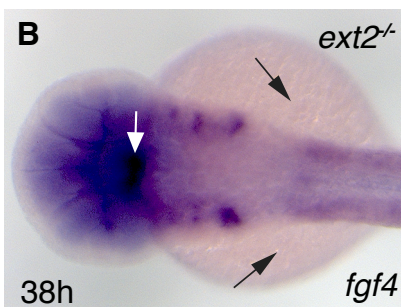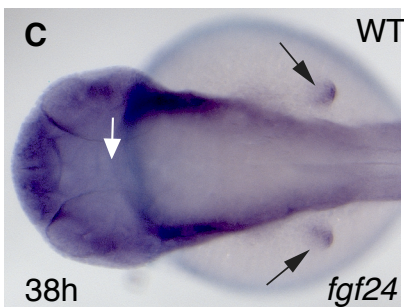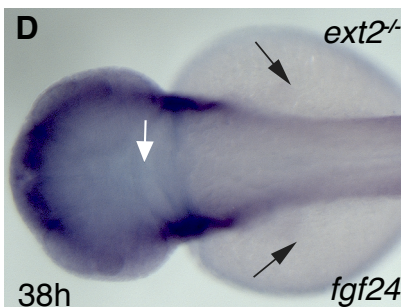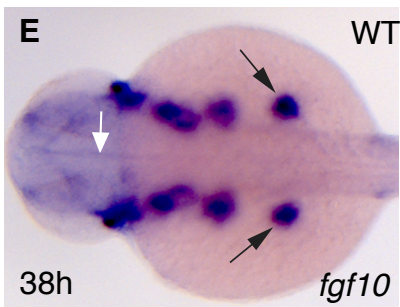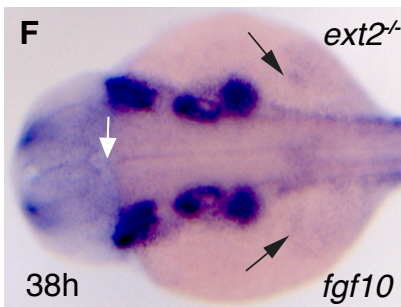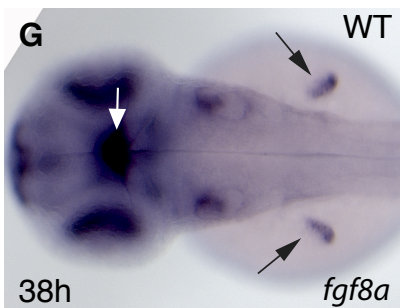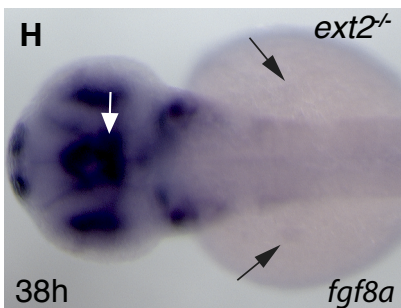

Supplement: Additional file 1 — Expression of fgf genes in ext2 mutants. Dorsal view of fgf4 (A-B), fg24 (C-D), fgf10 (E-F), fgf8 (G-H) expression in control embryos (left panels) and ext2 mutants (right panels) at 38 hpf. Note that comparable levels of all examined fgf genes are expressed at 38 hpf, with the exception of the developing pectoral fin. Black arrows label the presence (left panels) or absence (right panels) of developing pectoral fins. [file 1471-213X-11-53-S1.PDF]

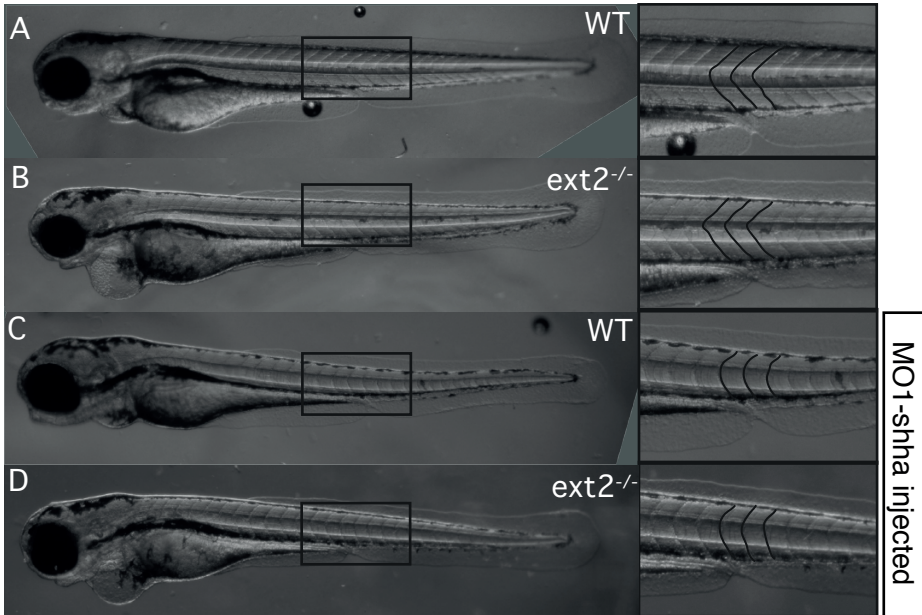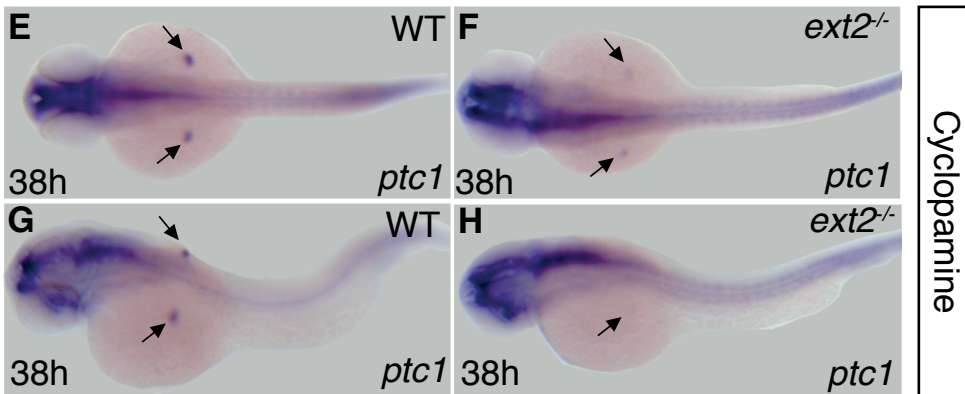

Supplement: Additional file 2 — Hh signaling is not sensitizised in ext2 mutants. Morpholino injection experiment (A-D). Lateral view of 3 dpf control (A, C) and ext2 mutants (B, D). In (C, D) 14 ng MO1-shha have been injected in the one-cell stage which has resulted in U-shaped somites in a portion of injected embryos (see result section). The shape of the somites is emphasized in the right panel (A-D). Cyclopamine treatment experiment (E-H). ptc1 expression in 38 hpf embryos subjected to 50 μM cyclopamine 32-38 hpf (E-H). Dorsal view (E, F) and dorsolateral view (G-H). WT embryos (E, G) and ext2 mutants (F-H). [file 1471-213X-11-53-S2.PDF]

Sibling

*ext2<sup>-/-</sup>* or *ext2<sup>-/-</sup>;shha<sup>+/-</sup>*

38h

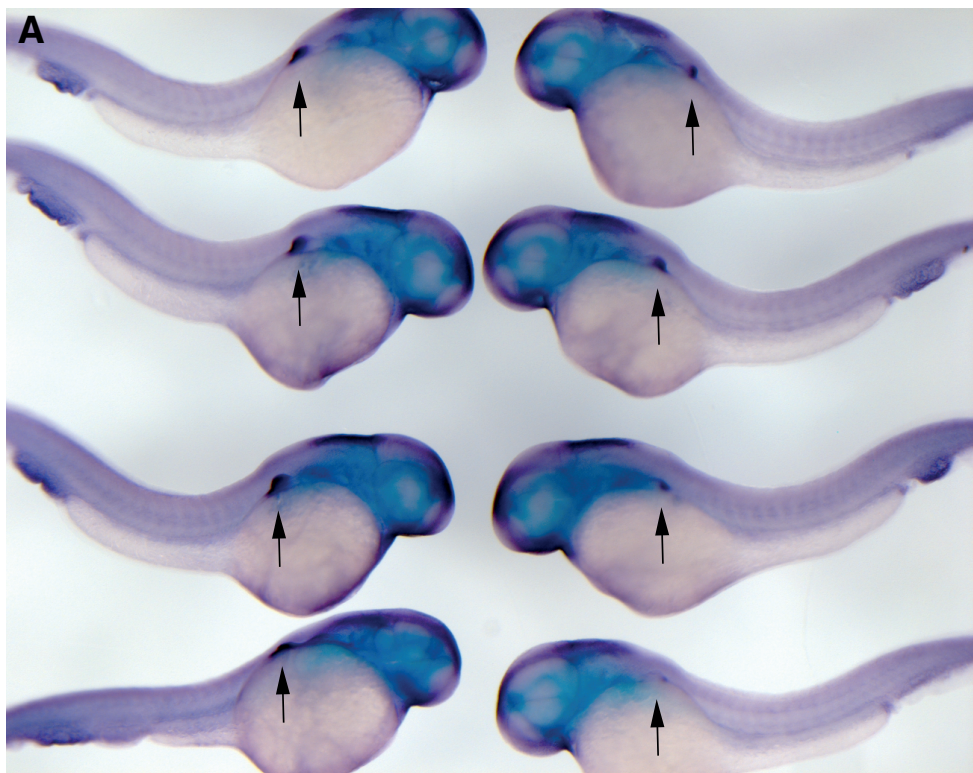

48h

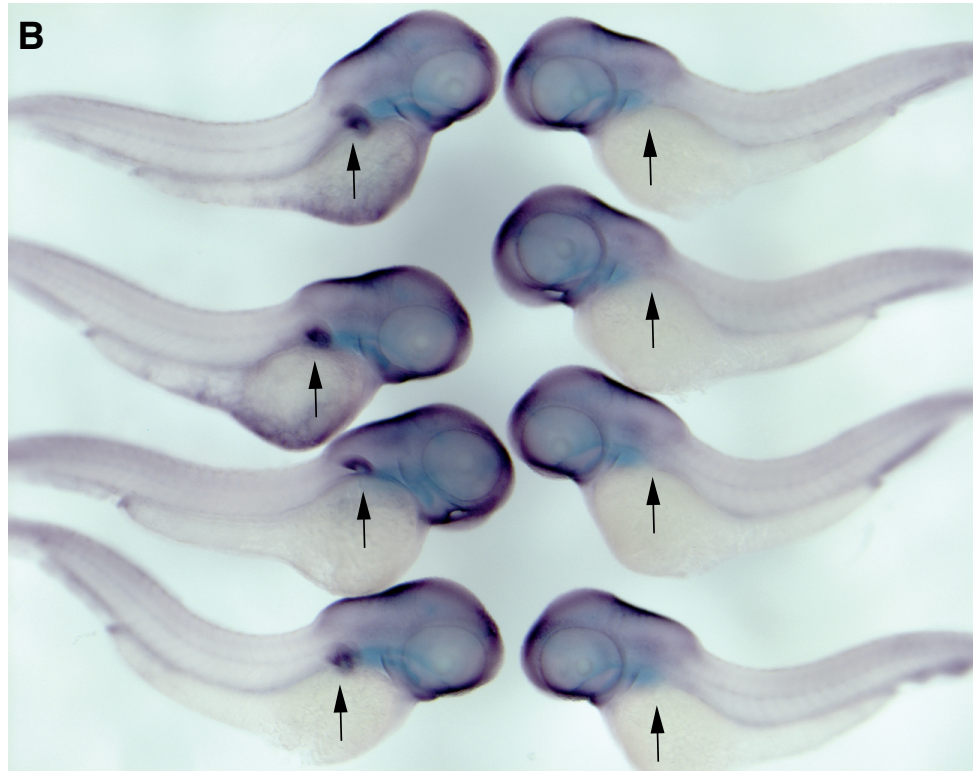

*ptc1*

Supplement: Additional file 3 — Hh signaling is not reduced in ext2-/-;shha+/- mutants. Genetic interaction experiment (A-B). Lateral view of ptc1 expression in 38 hpf (A) and 48 hpf (B) embryos from crossings of heterozygous ext2+/-;shha+/- double carriers with ext2+/- single carriers. Siblings (left row of embryos) and ext2-/-or ext2-/-;shha+/- mutants (right row of embryos). Arrows label the position of the pectoral fin. [file 1471-213X-11-53-S3.PDF]
